# CD4/CD8 Ratio could be predictor of burden hepatocellular carcinoma in Egyptian chronic hepatitis C after combined sofosbuvir and daclatasvir therapy

**DOI:** 10.4314/ahs.v23i1.22

**Published:** 2023-03

**Authors:** Nadia El Menshawy, Noha Hassan, Mohamed Khariza, Hala AlAshery, Monir Baghat, Rehab Ashour

**Affiliations:** 1 Mansoura University Faculty of Medicine, Clinical Pathology; 2 Mansoura University Faculty of Medicine, Pharmacy; 3 Mansoura University Faculty of Medicine, Internal Medicine

**Keywords:** HCV, DAAS Therapy, T helper, T Cytotoxic, natural killer cells, hepatocellular carcinoma

## Abstract

**Background:**

During the first years of the use of direct acting Hepatitis C antiviral drugs (DAAS), several studies reported a possible correlation between this new era of treatment and an increased risk of Hepatocellular carcinoma (HCC). Its development could possibly be favored by the changes in the immunological milieu and the different cellular behavior after eradication of HCV infection with them. For this reason, this study aimed to address the immunological effect of DAAS

**Subject & methods:**

Prospective paired -sample design, carried out on 90 naïve chronically infected HCV patients before and after receiving a combination therapy of sofosbuvir; at a dose of 400 mg once daily and daclatasvir; at a dose of 60 mg once daily for 12 weeks and follow up for one year. immunological tests including: total T cell count, T helper cell count, T cytotoxic cell count and natural killer cell count in peripheral blood through (CD3, CD3/CD4, CD3/CD8 and CD56 respectively) by Fluorochrome monoclonal antibodies labelled with specific dyes through Multiparameter, FACSCanto ™ II flow cytometer (Becton Dickinson, USA).

**Result:**

Concerning the immunological changes, total T cells (CD3+), Natural killer cells showed non-significant decrease at end of therapy while significant decrease in T helper cells (CD3+CD4+) T cytotoxic cells (CD3+CD8+) compared to pre-treatment value. Long follow up revealed 26.6% developed focal HCC, in more addition, multivariate analysis show CD4/CD8 ratio could be predictor as well as sex for early development of HCC after combined DAAS therapy.

**Conclusion:**

HCV treatment by DAAS produces significant decrease in T helper, T cytotoxic cells in CHC patients at the end of therapy. 26.6% developed focal HCC with independent CD4/CD8 predictor for burden malignancy. Further large extended population study is needed for clarify this concern.

## Introduction

HCV is a global health burden affecting approximately 71 million people worldwide with Egypt having the highest prevalence^1^. It causes annual deaths of approximately 399,000 which result mostly from complications^2^. HCV is a blood-borne virus transmitted mainly through infected blood and blood products^3^. It is a major cause of liver fibrosis, cirrhosis and hepatocellular carcinoma (in a rate of 1-7% each year). Hepatocellular carcinoma (HCC) is the third common cause of cancer death worldwide predisposing to one million deaths annually^4^–^5^.

Innate immunity plays the primary defense mechanism against HCV infection through Type I Interferon (IFN) production that makes the cells ready & promotes them to fight infection, checks viral replication, induces adaptive immune response in addition to stimulating certain cells as natural killer cells, dendritic cells & Kupffer cells^6^. NK cells represent an important arm of innate immunity that plays a critical role in HCV eradication. The stimulated NK cells recruit virus specific T cells, promote the antiviral immune response in the liver & eliminate virus infected hepatocytes directly through cytolytic mechanisms & indirectly by releasing cytokines such as INF-γ & TNF-α^7^.

HCV antigens are presented on MHC CLASS I & II molecules on the outer surface of the cell where they are identified by CD8+ & CD4+ T cells respectively. In more, HCV peptides liberated from damaged virus-infected hepatocytes are picked up by myeloid DCs that migrate to the draining lymph nodes (LNs) where they present HCV peptides on HLA class II particles with higher expression of CD80 & CD86 costimulatory particles that react with & stimulate HCV specific T helper cells^8^. These cells induce DCS maturation & greater expression of CD40 ligand & TNF-α. In turn, these mature DCS induce T cell activation by over expression of their surface markers. IL-12 has a significant role in enhancing INF-γ secretion from activated T cells & subsequently promotes the evolution of Th1 immune response that characterizes cytotoxic T lymphocyte (CTL) activation. The activated CTLs secrete perforin, granzyme & TNF-α or express FAS ligand & directly invade virus infected liver cells Activated CTLs cause death of HCV-infected cells & also play a role in virus control through a non-cytolytic mechanism by releasing cytokines such as INF-γ, INF-α/β & TNF-α that promote an antiviral status in host cells. Also, this makes uninfected cells resistant to infection & hinders HCV replication^9^.

Interferon-based therapy was the core therapy of chronic HCV infection with cure rate of about 50% & with many side effects. This therapy was recently replaced by new drugs called direct acting antivirals (DAAS) with high cure rate indicated by achieving sustained virological response (SVR) in more than 90% of patients with shorter duration of treatment & little adverse effects than interferon-based therapy. These DAAS target viral replication through affecting viral proteins essential for it such as ns3/4a inhibitors, ns5a inhibitors & ns5b inhibitors^10^–^11^. This high SVR achieved by DAAS was expected to reduce the risk of HCC. However, the effect of treatment by DAAS on recurrence & occurrence of new HCC in patients with cirrhosis after successful treatment is conflicting & controversial^12^. Several studies reported increased incidence of HCC recurrence, de novo HCC & aggressive relapse of HCC during & after receiving DAAS treatment. High recurrence of 27% raised the debate that DAAS may be incriminated in predisposition of HCC recurrence or new occurrence^13^–^14^. Affection of immune system after viral eradication by DAAS may be a contributing factor to HCC recurrence or de novo occurrence^15^.

For this reason, the current study aimed to evaluate certain immune markers (total T cells, T helper cells, T cytotoxic cells and natural killer cells) in chronic HCV patients treated with sofosbuvir and daclatasvir before therapy and after 12 weeks and 48 weeks of therapy to detect the effect of this combination therapy of direct acting antiviral therapy on the immune status of these patients.

## Patients and Methods

This study was paired -sample design, one arm prospective carried out on 90 naïve chronically infected HCV patients before and after receiving a combination therapy of sofosbuvir; at a dose of 400 mg once daily and daclatasvir; at a dose of 60 mg once daily for 12 weeks and 48 weeks follow up. Those 66 patients were males (73.3%) & 24 females (26.7%) with mean age of 44.27 ± 8.48. The patients were collected from hepatology outpatient clinic of Mansoura Specialized Internal Medicine Hospital during the period from October 2017 to October 2018.

The study was conducted according to guidelines for good clinical practice and approved by the ethics committee of Faculty of Medicine, Mansoura University. The purpose and procedures included in the study were explained to all participants and informed consent was obtained from all of them. Patients were selected according to the following criteria. Age: more than 1 8, sex: both male and female & patients with no contradication to DAAS therapy. While Patients with chronic renal failure, patients with Human immunodeficiency virus (HIV) infection, patients with hepatitis B virus (HBV) infection & patients with hepatocellular carcinoma (HCC) were excluded. All patients were subjected to the following: Full history taking, complete clinical examination with the following investigations done before receiving any treatment and after 12- week therapy with sofosbuvir and daclatasvir and follow up for one year after therapy.

Quantitative assessment of HCV RNA by RT-PCR using COBAS TaqMan HCV test (version 2.0; Roche Molecular Systems, Pleasanton, CA) with a lower limit of quantification (LLOQ) of 25 IU/µl, liver function tests (serum albumin, total bilirubin, alanine aminotransferase “ALT” and aspartate aminotransferase “AST” creatinine, fasting blood glucose ) (colorimetric method, Cobas C311 automatic analyzer, Germany), complete blood count (CBC) (automated cell counter, sysmex Kx21, Germany), international normalized ratio (INR) (Quatron A4, Germany), alpha feto protein (AFP)&TSH (chemiluminescence, Cobas 411, Germany) (before treatment only), Germany) anti-nuclear antibodies (ANA) (ELISA,anti-bilharzial antibodies by indirect haemagglutination (IHA) test (Schistosomiasis Fumose, Laboratories Fumouze Diagnostics, France), pregnancy test in females of childbearing period (before treatment only), hepatitis B surface antigen (ELISA), abdominal ultrasound, ECG in old aged patients & immunological tests including: total T cell count, T helper cell count, T cytotoxic cell count and natural killer cell count in peripheral blood smear as indication for immune status through detection of surface markers on these cells (CD3, CD3/CD4, CD3/CD8 and CD56 respectively) by monoclonal antibodies labelled with specific dyes through Multiparameter, 8 fluoro-coloured, 3 laser, FACSCanto ™ II flow cytometer (Becton Dickinson, USA SN: V33896202133)^16^

## Methods

### Sampling

Venous blood sample 2 ml were extracted from each patient without stasis to purple vacuum tubes containing EDTA transport immediately to be processed within 4 hours at flow cytometry unit of Oncology Center, Mansoura University (OCMU).

Cells are conjugated with specific fluorescent labelled monoclonal antibodies and then acquired on FACS Canto II (BD bioscience, San Jose, CA) and analysed using flow software (BD Bioscience). Fluorescein isothiocyanate (FITC)-labelled anti-human CD3 mAb (clone: HIT3a, Catalog Number: 300328), allophycocyanin (APC)-labelled anti-human CD4 mAb (clone: MT310, Catalog Number: C7226), phycoerythrin (PE)-labelled anti-human CD8 mAb (clone: RPA-T8, Catalog Number: 555367), phycoerythrin (PE)-labelled anti-human CD56 mAb (clone: MEM-188, Catalog Number: 304628). All were purchased from Biolegend Inc, San Diego, CA. They were used at concentrations recommended by their manufacturers.

Blood was distributed in three tubes (about 200 µl in each tube calculated according to the equation 1000/WBC count). One tube contained only blood (auto) to adjust auto fluorscent background before analysis, second tube contained blood with 5 µl anti CD3, CD4 and CD8 mAbs labelled with FITC, APC and PE respectively and the third tube contained blood with 5 µl PE-labelled anti CD56 mAb. Blood was then incubated with fluorochrome-conjugated monoclonal antibodies for 15 minutes in the dark at room temperature. Then 2ml of ammonium chloride were added to each tube to induce lysis of red blood cells. The samples were then vortexed and incubated in the dark for 15 minutes followed also by vortex. Centrifugation at a speed of 3000 RPM was then done for 5 minutes and supernatant was discarded, then the pellet was formed. This was followed by wash with 0.5 ml or 1 ml of phosphate buffered saline for two times or more till clearance of red blood cells. Vortex was then done followed by centrifugation followed by wash until all blood around the pellet was cleared to get rid of debris and 500 µl PPS were left on the specimen. Eight-colour flow cytometry was then done using FACSCanto (Becton Dickinson and company, BD Bioscience, San Jose, CA 95131 USA) and data were analysed using FACS ADIVIA software to detect surface marker expression through flourochrome-labeled monoclonal antibodies. Gating strategy was done using bright CD45 mononuclear cells with counting total CD3, absolute count of CD4 and CD8 from total CD3 with calculating CD4/CD8 ratio in addition to counting natural killer cells which express CD56.

By plotting CD3 versus CD4, we can get total T cell (CD3^+^) count and T helper cell count (CD3^+^ CD4^+^) and by plotting CD3 versus CD8, we can get also total T cell count and T cytotoxic cell count (CD3^+^ CD8^+^). Also, by plotting CD4 versus CD8, we can obtain CD4/CD8 ratio. the count of natural killer cells through (CD56^+^).

### Sample size

Sample size was calculated using PASS software (version 2008). Estimation relied upon a previous study by17.

### Statistical analysis

Data were entered and analyzed using IBM-SPSS software (version 25). Qualitative data were expressed as frequency and percentage. Quantitative data were initially tested for normality using Shapiro-Wilk's test with data being normally distributed if p>0.050. Quantitative data were expressed as mean ± standard deviation (SD) if normally distributed or median and interquartile range (IQR) if no Paired-Samples t-test was used if data were normally distributed in both readings. The non-parametric alternative Wilcoxon signed ranks test was used if not. Repeated-measures ANOVA test was used if data were normally distributed in all readings. results were considered as statistically significant if p value ≤ 0.050.

## Results

The demographic, clinical characterization and basic laboratory data are shown in tables (1-3).

**Table 1:** Demographic and clinical distribution of the studied case

Variable		Distribution
**Age (years)**	44.27 ± 8.48	
**Gender (Male)**	66	73.3
**Female**	24	26.7
**Weight (kg)**		90.9 ± 16.03
**Height (m)**		1.68 ± 0.11
**BMI (kg/m2)**		32.7 ± 7.12

Data was presented as mean, SD, Percentage. BMI; Body Mass Index.

**Table 2:** Basic laboratory investigation for chronic hepatitis C assessment

Variable	Baseline value
**HCV RNA (IU/ml)**	721426 (114480 - 1134208)
**AFP (IU/L)**	3.2 (1.9 - 8.4)
**TSH (µIU/ml)**	1.8 ± 1.155
**FBG (mg/dl)**	89 ± 9.99
**FIB4 [(Age×AST) /** **(Platelet count×ALT)]**	0.93 ± 0.21

Data are represented as mean ± standard deviation or median (inter-quartile range). Abbreviations: AFP, alpha feto protein. TSH, thyroid stimulating hormone. FBG, fasting blood glucose.FIB-4, Fibrosis-score 4

**Table 3:** Clinical characterization of studies case

Variable	Frequency	Percentage
**Occupation:** **manual** **mental**	84 6	93.3% 6.7%
**Smoking** **Non smoker** **Smoker**	84 6	93.3% 6.7%
**Hypertension** **Not hypertensive** **Hypertensive**	60 30	66.7% 33.3%
**Residency** **Rural** **Urban**	72 18	80% 20%
**IHA-Bilharziasis** **Negative** **Positive**	54 36	60% 40%

Data was presented as frequency and percentage.

IHA-Bilharziasis: Indirect Hemagglutination test for bilharzial antigen detection.

The changes in the basic biochemical and hematological parameter with 3 months follow up.

The statistical analysis revealed significant decrease in hemoglobin level; Platelet's count; ALT; AST; serum albumin: INR and no change in WBCs count; Serum bilirubin: serum creatinine (table 4)

**Table 4:** Basic biochemical and hematological parameter with 3 months follow up

Variable	Baseline	At 1-Month	At 2-Months	At 3-Months	P value
**ALT** **(IU/L)**	35 (23–119)	21 (15.7–25)	18 (15–22)	18 (16–21.7)	0.008*
**AST** **(IU/L)**	34 (28–97)	21 (16-–7.3)	21(17–22)	19 (16–22)	0.037*
**S. albumin** **(gm/dl)**	4.42± 0.4	4.43± 0.39	4.46± 0.23	4.31± 0.36	0.006**
**Total bilirubin** **(mg/dl)**	0.65± 12	0.65±.14	0.65±.1	0.65±.12	0.79*
**WBC** **(10^3^/mm^3^)**	7.7±2.15	8.01±1.84	7.9±1.52	7.84±2.27	0.135**
**Hemoglobin** **(gm/dl)**	13.26±2.16	12.97±2.07	12.51±2.13	12.46±1.82	0.001**
**Platelets (10^3^/mm^3^)**	308±80.8	270±74.9	242.47±37.48	265.73±55.18	0.013**
**INR**	1.03±0.5	1.07±.06	1.06±0.8	1.07±0.09	0.005*
**S. creatinine (mg/dl)**	0.72±0.7	0.79±.18	.86±31	0.81±.13	0.199*

Data are represented as mean ± standard deviation or median (inter-quartile range).

**Figure 1:**
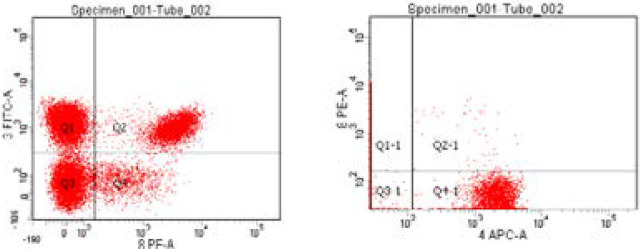
Dot plot represents CD3-FITC versus CD8-PE in one histogram and CD4-APC versus CD8-PE in another histogram.

**Figure 2:**
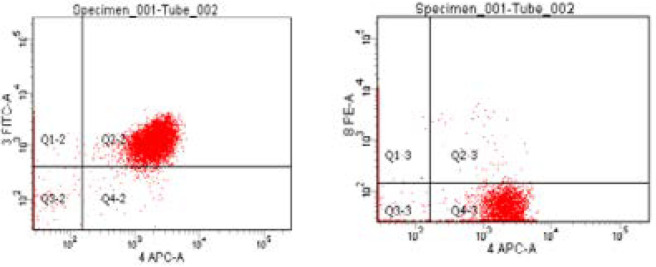
Dot plot represents CD3-FITC versus CD4-APC in one histogram and CD4-APC versus CD8-

**Figure 3:**
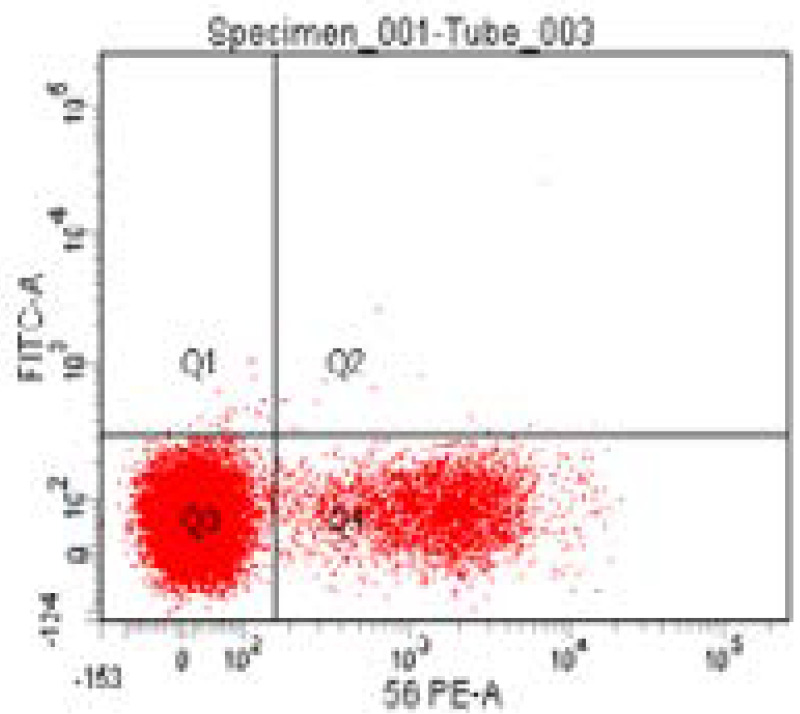
Dot plot represents CD56-PE versus FITC.

Impact of Drug on patients' Immunological criteria of the studied cases: At 3 months no significant changes total CD3; CD4/CD8 ratio; NK while significant reduction was observed in T-helper cells CD3/CD4 and T-cytotoxic cells CD3/CD8 in study cases (table 5 & figures 1,2,3) Long follow up for 48 weeks revealed significant characterization of developed HCC in study group (table 6).

**Table 5:** Immunological tests of the studied cases

Variable	Baseline	At 3month	P value
**Total T cells** **CD3**	61.43±17.22	60.01±13.37	0.48
**T helper cells** **CD3/CD4**	36.68±9.23	33.85±10.9	0.001
**T cytotoxic cells** **CD3/CD8**	22.01±7.66	20.74±6.98	0.02
**Natural killer cells** **CD56**	12.77±7.66	12.59±6.98	0.76
**CD4/CD8 ratio**	1.58±0.55	1.49±0.50	0.034**

Data are represented as mean± standard deviation.

P values with ** are measured by paired sample T test

**Table 6:** Clinical characterization of HCC versus good remission in study population

		follow up		P
		Good	HCC	
**Age**		**46.09±7.96**	**39.25±7.96**	0.001*
**ALT (12M)**		**18.00(16.00–21.00)**	**18.50(13.50–23.00)**	0.74
**AST (12M)**		**20.20(16.00–23.40)**	**18.50(17.00–19.50)**	0.1
**Albumin (12M)**		**4.25±.33**	**4.50±.38**	0.003*
**T_bilirubin (12M)**		**.63±.14**	**.71±.02**	0.005*
**WBCs(12M)**		**7.89±1.37**	**7.70±3.82**	0.72
**Hb(12M)**		**12.22±1.85**	**13.12±1.57**	0.036*
**PLT (12M)**		**277.73±52.67**	**232.75±48.93**	<0.001*
**INR (12M)**		**1.06±.07**	**1.11±.11**	0.017*
**creatinine(12M)**		**.80±.11**	**.86±.19**	0.08
**CD4/CD8 ratio(12M)**		**1.62±.42**	**1.03±.30**	<0.001*
**A-feto pro (12M)**		**3.90(2.90–4.60)**	**105.00(80.00–120.50)**	<0.001*
**Sex**	**Female**	**6(9.1%)**	**18(75.0%)**	<0.001*
	**Male**	**60(90.9%)**	**6(25.0%)**	
**Occupation**	**No**	**66(100.0%)**	**18(75.0%)**	<0.001*
	**Yes**	**0(0%)**	**6(25.0%)**	
**Smoking**	**N**	**66(100.0%)**	**18(75.0%)**	<0.001*
	**P**	**0(0%)**	**6(25.0%)**	
**HTN**	**N**	**54(81.8%)**	**6(25.0%)**	<0.001*
	**P**	**12(18.2%)**	**18(75.0%)**	
**Residency**	**rural**	**54(81.8%)**	**18(75.0%)**	0.55
	**urban**	**12(18.2%)**	**6(25.0%)**	
**IHA_bilharziasis**	**N**	**42(63.6%)**	**12(50.0%)**	0.33
	**P**	**24(36.4%)**	**12(50.0%)**	

Data expressed as mean±SD & Median (IQR), frequency (number, percent)

SD: standard deviation, IQR: interquartile range

P: Probability

Test used: Student's t-test (Paired) for data expressed as mean SD & Wilcoxon signed rank test for data expressed as Median (IQR), Pearson's chi square or fisher-exact test for data expressed as frequency

Univariate regression analysis in study group shows different predictor for hepatocellular carcinoma as age, albumin, Hb, platelet, INR, CD4/CD8 ratio, Sex, hypertension while forward stepwise multivariate regression analysis revealed only CD4/CD8 ratio (P 0.004 OR 0.003 95% CI 00-0.165 and sex P 0.001 OR 0.017, 95% CI 0.002-0.16 are the most independent prognostic predictor for HCC as show in tables (7&8).

**Table 7:** Univariate regression analysis in study group

Univariate			
	P value	OR	95 % CI
Age	0.001	.899	.843-.960
ALT	0.386	.944	.829–1.075
AST	0.06	.872	.756–1.006
Albumin	0.004	9.764	2.034–46.881
WBCs	0.722	.962	.779–1.189
Hb	0.041	1.375	1.013–1.865
Platelet	0.002	.981	.970-.993
INR	0.021	4.857	2.512–9.913
CD4/CD8 ratio	0.001	0.027	0.005-,131
Sex	0.001	0.033	0.010-.116
HTN	0.001	13.5	4.423–41.200
Residency	0.476	1.524	0.491–4.568
Bilharzias	0.245	1.75	0.681–4.499

**Abbreviations**: ALT, Alanine amino transferase. AST, Aspartate aminotransferase. WBC, white blood cells. INR, International normalization ratio, HTN, Hypertension

**Table 8:** Multivariate regression analysis (Forward step wise)

	P	OR	95% CI
**CD4/CD8 ratio**	***0.004***	0.003	0–0.156
**Sex**	***0.001***	**0.017**	**0.002–0.16**

## Discussion

One of the most serious global infections is chronic hepatitis C virus (HCV) that affects about 71 million individuals worldwide, representing 1.1% of the world's entire population^18^. Globally, around 400,000 deaths and ∼3–4 million new infections are caused by HCV every year^19^. HCV increases the susceptibility of patients at risk to develop cirrhosis, portal hypertension, liver failure and also hepatocellular carcinoma besides being the leading indication for liver transplantation in Western countries. Therefore, HCV eradication is an important aim to manage these complications and stop further spread of HCV disease^20^.

The prevalence of HCV antibody is still high in Egypt, measured at 10.0% for the age group between 15- and 59-year-old, with genotype 4 (GT4) representing about 90% of infections with predominating subtype 4a^21^.

HCV therapy has been revolutionized in the few recent years by the appearance of direct-acting antiviral agents (DAAS). These greatly effective, very well-tolerated DAAS of several classes have largely substituted PEGIFN and RBV. The advantage of these new regimens is that; they produce greater sustained virologic response (SVR) rates with more possibility of adherence to therapy in addition to much fewer side effects and much less diminished health-related quality of life during therapy^22^. The elevated rates of SVR attained in Chronic hepatitis C(CHC) patients treated with DAAS were expected to raise the hope of a significant reduction in HCC occurrence and recurrence. Unexpectedly, patients who cleared HCV with DAAS have been found to encounter increased aggressiveness and elevated rates of HCC recurrence (28% and 29% respectively) after a complete response to resection or local ablation within only 6 months of therapy13-14. On the other hand, increased risk of HCC recurrence after DAAS treatment in CHC patients after receiving curative cancer treatments couldn't be revealed by three independent prospective French cohorts^23^. These contradictory results have increased commentaries and criticism about this controversial issu^24^. Despite the unclear effects of DAAS therapy on the rate of HCC occurrence or recurrence, it would be important to study the immunological alterations in CHC patients treated with DAAS^25^–^26^.

Sofosbuvir is the principal DAA in all published reports of DAAS related HCC. In spite of that, the supposed relation between DAAS in general, sofosbuvir or sofosbuvir linked metabolites and carcinogenesis requires more analysis. Although, multiple theories were hypothesized to clarify this supposed linkage, none of them had a strong proof of concept^13^.

This study aimed to detect the effect of sofosbuvir and daclatasvir combination therapy of DAAS on the immune status in chronic hepatitis c patients follow up of patients for one year and could be predictor of burden Hepatocellular carcinoma.

This Egyptian study was carried out on 90 naive chronically infected hepatitis C patients, treated by combined DAAS antiviral therapy for 12 weeks then follow up for one year.

The present study revealed significant reduction of both ALT, AST at the end of completion therapy, this finding similar to same observation on similar population with both combined therapy^27^–^28^. As HCV is believed to be non-cytopathic, prompt depression, either quantitatively or qualitatively, of intrahepatic cytotoxic inflammatory cells might be the cause of prompt AST/ALT normalization^29^.

No significant change was observed in serum albumin level between the end of therapy and pre-treatment period. This was similar to the results conducted by^27^–^30^. While disagree with study by Fayed et al. showed an increase in albumin level^31^. The difference between our study and this study may be due to the possibility that longer duration may be needed for the improvement in serum albumin level and consequently the synthetic function of the liver as reported by Maruoka et al. who noted that over the first two years after combined interferon treatment, serum albumin level increased gradually then plateaued^32^. This improvement reinforces the belief that intra-hepatic inflammation directly, participate in reduced synthetic ability of the liver and that ameliorating inflammation can reconstruct liver function to some degree^33^.

Total bilirubin level at the termination of therapy was not found at statistically significant change from pre-treatment level. This was also in agreement with the results obtained^34^–^35^.

As regarding renal function assessment by Serum creatinine had non-significantly different level at end of therapy compared to pre-treatment level. This was also in agreement with^27^–^34^. while in agreement with study by Sulkowski et al. that assessed sofosbuvir and daclatasvir in untreated patients and previously treated patients who failed telaprevir and bocebrevir treatment showed increase of creatinine at the end of therapy^36^. This difference in observation may be related to the effect load of drug therapy used in protocol regiment on renal function. Considering the hematological parameters, White blood cell count (WBC) wasn't significantly changed at end of therapy compared to pre-treatment. This finding similar to^31^–^37^. In contrast, the study by Elsharkawy et al. showed significant reduction of WBCs at the end of treatment^30^. Hemoglobin level showed non-significant change at the end of therapy, similar to observation of^34^–^38^ In contrast, there was significant decrease of hemoglobin at the end of therapy compared to pre-treatment^36^. Platelet count decreased significantly at 2 months of therapy compared to pre-treatment level. The level increased at end of treatment but was still lower than the pre-treatment level. Platelet count decreased at the termination of therapy when compared to healthy subjects in the study of^38^ while there was non-significant decrease in platelet count 3 months post-therapy compared to pre-treatment level in the study of^37^ and at the end of therapy compared to baseline in the study of^31^. In contrast, median platelet count increased from baseline to the end of follow up in the study conducted by Giannini et al. using sofosbuvir based regimens with or without ribavirin for 12 or 24 weeks^39^.

International Normalized Ratio (INR) exhibited non-significant difference at end of therapy compared to pre-treatment value. Non modification in median INR at the various study time points was also observed in the study of Giannini et al^39^. While there was a median reduction in INR by 0.13 points in the study of Belli et al. that included 103 patients treated with sofosbuvir based regimens only for 12, 24 or 48 weeks^40^. This difference could be explained by different follow up periods. Longer periods of follow up may be required to get significant results.

Concerning the immunological changes, total T cells (CD3^+^) Showed non-significant decrease at end of therapy compared to pre-treatment value. Similar finding to study by Stevenson et al. showed non-significant difference in CD3^+^ lymphocytes at any sample point compared to pretreatment.^41^ While disagree with study by Burchill et al. that assessed 19 treatment-naive cases having chronic HCV genotype 1a/1b who were treated with an IFN and ribavirin-free regimen composed of daclatasvir (NS5A inhibitor), asunaprevir (NS3 protease inhibitor) and BMS-791325 (non-nucleoside NS5B inhibitor) showed restoration in the percentage of total T lymphocyte after DAAS treatment; however, it is unlikely for the DAA regimen utilized in this study to become a mainstream therapy for HCV because the FDA new drug application for asunaprevir was lately withdrawn by BMS^42^.

T helper cells (CD3^+^ CD4^+^) showed statistically significant decrease at end of treatment compared to pre-treatment value. While in agreement with study by Li et al. showed no significant difference in the frequency of CD4 T cells between CHC patients and healthy controls before or after INF free therapy^43^. This discrepancy due to different study protocols, the percentage of CD4 T cells increased after therapy in the study of Burchill et al.^42^

T cytotoxic cells (CD3^+^ CD8^+^) showed statistically significant decrease at the termination of therapy when compared to pre-treatment value. Similar to study by Pereira et al. showed decreased CD8 T cell count at the termination of therapy in comparison to healthy subjects^38^, while the study done by Burchill et al. although resulted in high CD4 T cells percentage, the percentage of CD8 T cells did not increase in peripheral blood^42^. In contrast, the percentage of CD8 T cells gradually increased to the level of healthy controls by the end of DAAS therapy in the study done by Li et al^43^. While study by Meissner et al. that revealed a remarkable early rise in the peripheral lymphocyte count (CD4 and CD8) 1-2 weeks' post-treatment, such increase is not consistently maintained throughout the course of therapy. This increase noticed 1-2 weeks following the start of therapy correlated with decline in liver transaminase levels and could indicate both decreased migration of peripheral cells to the liver resulting from altered chemotaxis as well as potential exit of tissue-resident lymphocytes from the liver. This difference may be related to changes in time of specimen collection, gating strategies and sample sizes^33^.

Subsequently, CD4/CD8 ratio also showed significant difference at end of treatment compared to pre-treatment value. This finding did not match to study by Telatin et al. noted that after nearly 2 years from the treatment end and viral clearance, T lymphocytes abnormalities are partially restored^44^.

Natural killer cells expressed non-significant decrease at the termination of therapy in comparison to pre-treatment level. Similar finding with^29^–^45^. While dis matched with Li et al who observed he percentage of NK cells increased to normal level of healthy control at 12 weeks of therapy and remained at normal level at 24 weeks in the study of Li et al43. Different factors may impact the dynamic alterations in NK cells during DAAS treatment such as different HCV genotypes, different DAAS regimens and different races. Additionally, NK cell recovery may take a long period and also recovery of NK cells after the influence from DAAs is relieved cannot be achieved immediately^46^.

Longer follow up for study 48 weeks show 24/90 (26.6%) developed HCC as confirmed by α fetoprotein and ultrasound imaging, condition of those patients shows statistically significant changes from good condition after therapy, this cases more frequent in female and hypertensive patients. Regression analysis by univariate test Univariate regression analysis in study group show different predictor for hepatocellular carcinoma as age, albumin, Hb, platelet, INR, CD4/CD8 ratio, Sex, hypertension while forward stepwise multivariant regression analysis revealed only CD4/CD8 ratio (p 0.004 OR 0.003 95% CI 00-0.165 and sex 0.001 OR 0.017, 95% CI 0.002-0.16 are the most independent prognostic predictor for HCC.

The emergence of HCC was linked, to baseline risk factors including advanced fibrosis stage, HBV co-infection or age. Also, it was suggested by another hypothesis that DAAS promote dysregulation of immune surveillance mechanisms after the very rapid viral eradication; this hypothesis has been promoted by several studies. The reconstitution of innate immunity with the downregulation of type II and III IFNs receptors and IFN-stimulated genes. A decreased stimulation of IFN may subsequently permit the growth of neoplastic cells as the INF has anti-angiogenic and anti-proliferative characteristics that DAAs lack. Also, the reduction in the cytotoxic activity of NK cells in the liver is one of the immune system changes that have been described after HCV eradication, which supports a more rapid progression of HCC foci^29^. This finding was confirmed by an interesting study carried out by Monto and his colleagues who observed that NK cell inhibitory KIR/HLA types were found in all 11 patients who developed HCC after second generation DAA indicating the genetic based impaired immune-surveillance capacity in those patients^47^. MicroRNA (miRNA) 122 that is the major miRNA in hepatocytes could be related to another potential mechanism. As re it acts as a tumor suppressor gene in HCC^48^. Interestingly, down regulation of miRNA 122 after achieving SVR through DAA therapy was observed; this may contribute to an elevated risk of HCC recurrence^49^. Eventually, another significant study by Villani et al. revealed that the level of vascular endothelial growth factor becomes significantly elevated during treatment with DAA and remains elevated for 3 months after treatment cessation that may finally lead to the occurrence/recurrence of HCC^50^.Unique identification of T-cell population in HCC could help in targeted therapy or patient may benefit from immunotherapy later on, CAR-T cell in future could destroy malignant cells^51^.

Limitations of the present study include limited scope of research population. Also, we didn't assess the hepatic immune changes and the phenotypic changes after DAAS therapy. So, further studies with large sample size, prolonged follow up for 2 years or more and wide scope of research with different genotypes are needed to confirm the results of the present work and to investigate different changes in peripheral immunity in correlation with hepatic immunity and phenotypic immune changes.

## Conclusion

HCV treatment by DAAS produces non-significant decrease in the count of total T cells, natural cells, while significant reduction in T helper cells, T cytotoxic cells, CD4/CD8 ratio in peripheral blood of CHC patients at the end of therapy. longer periods of follow up revealed26.6% developed HCC. Regression analysis revealed significant CD4/C8 ratio as early predictor of HCC after DAAs therapy.
